# Chlorogenic Acid Intravesical Therapy Changes Acute Voiding Behavior of Systemic Lipopolysaccharide Inflammation-Induced Cystitis Bladder in Mice

**DOI:** 10.3390/toxics12040239

**Published:** 2024-03-25

**Authors:** Chung-Hsin Yeh, Chellappan Praveen Rajneesh, Chun-Hou Liao, Wen-Chen You, Kuo-Chiang Chen, Yi-No Wu, Han-Sun Chiang

**Affiliations:** 1 Division of Urology, Department of Surgery, Shin Kong Wu Ho-Su Memorial Hospital, Taipei City 111045, Taiwan; m000732@ms.skh.org.tw; 2 School of Medicine, College of Medicine, Fu Jen Catholic University, New Taipei City 242062, Taiwan; praveenrajneesh@gmail.com (C.P.R.); liaoch22@gmail.com (C.-H.L.); tiffanyyou8669@gmail.com (W.-C.Y.); kkhk88@gmail.com (K.-C.C.); 3 Division of Urology, Department of Surgery, Cardinal Tien Hospital, New Taipei City 231403, Taiwan; 4 Department of Urology, Cathay General Hospital, Taipei City 106438, Taiwan; 5 Graduate Institute of Biomedical and Pharmaceutical Science, College of Medicine, Fu Jen Catholic University, New Taipei City 242062, Taiwan; 6 Department of Urology, Fu Jen Catholic University Hospital, New Taipei City 243089, Taiwan

**Keywords:** bladder pain syndrome, chlorogenic acid, interstitial cystitis, lipopolysaccharide

## Abstract

This study explores the potential efficacy of chlorogenic acid (CGA) in mitigating lipopolysaccharide (LPS)-induced cystitis in a mice model. C57BL/6J mice were divided into four groups: normal control (NC), LPS, LPS + low CGA, and LPS + high CGA. Evaluation methods included cystometrogram (CMG), histopathological, western blot, and immunohistological analysis. In the LPS group, CMG revealed abnormal voiding behavior with increased micturition pressure, voided volume (VV), and decreased voided frequency. Low CGA treatment in LPS mice demonstrated improved micturition pressure and inter-contraction intervals (ICI). However, high CGA treatment exhibited prolonged ICI and increased VV, suggesting potential adverse effects. Histological analysis of LPS-treated mice displayed bladder inflammation and interstitial edema. Low CGA treatment reduced interstitial edema and bladder inflammation, confirmed by Masson’s trichrome staining. Western blotting revealed increased cytokeratin 20 (K20) expression in the low CGA group, indicating structural abnormalities in the bladder umbrella layer after LPS administration. In conclusion, low CGA treatment positively impacted voiding behavior and decreased bladder edema and inflammation in the LPS-induced cystitis mice model, suggesting its potential as a supplement for inflammation cystitis prevention. However, high CGA treatment exhibited adverse effects, emphasizing the importance of dosage considerations in therapeutic applications.

## 1. Introduction

Interstitial cystitis (IC), also known as bladder pain syndrome (BPS), is a chronic and inflammatory disorder that significantly affects the quality of life. It is characterized by sensations of pain, pressure, or discomfort centered around the urinary bladder [1]. This sensation is often accompanied by urgency and frequent urination, persisting for more than 6 weeks, even in the absence of infection or pelvic irregularities. IC is marked by specific bladder observations, such as glomerulations or Hunner ulcers, along with histological evidence of submucosal inflammation or a certain quantity of mast cells [2]. The symptoms of IC arise from bladder urothelial dysfunction, neurogenic inflammation, and neuropathic pain. This inflammation leads to the thinning of the protective glycosaminoglycan layer, resulting in increased nerve sensitivity and urination frequency [3].

The treatment landscape for IC remains inadequately defined, lacking universally effective therapeutic measures. New and emerging IC therapies are needed, especially for patients who fail to respond to currently available treatments. The mechanisms underlying IC remain theoretical and uncertain, prompting researchers to explore treatment options using animal models [4,5]. The examination of pain and inflammation mechanisms linked to cystitis has conventionally employed external stimuli to induce IC in rodent models. Lipopolysaccharide (LPS), a common agent for inducing detrusor smooth muscle inflammation, is frequently utilized to replicate IC in animal models, facilitating the study of tissue fibrosis progression [6,7]. In addition to the endotoxin LPS, a combination of protamine sulfate can establish a more effective bladder injury mouse model that effectively simulates IC [8]. In accordance with our recent study [3], we have established that the B6 (C57BL/6J) mice strain is more suitable for IC and cystometrogram (CMG) investigations. Intraperitoneal LPS injection induces inflammation in mice, primarily characterized by bladder wall edema [9].

Chlorogenic acid (CGA), a prominent phenolic acid abundant in dietary staples like coffee and tea [10], has shown remarkable potential in hepatoprotection against LPS or chemical-induced hepatic damage [11]. This multifaceted compound enhances endothelial function and ameliorates insulin resistance [12]. Bhandarkar et al. [12], supported by in vitro research, demonstrated that CGA effectively combats endothelial dysfunction and augments vascular endothelial function through the release of nitric oxide and reduction in homocysteine levels [13]. Additionally, the protective nature of CGA against diabetic nephropathy is attributed to its modulation of the Nrf2/HO-1 and NF-ĸB pathways, resulting in a notable reduction in oxidative stress and inflammation [14]. Furthermore, CGA also exhibits the potential to ameliorate inflammation associated with L-arginine-induced pancreatitis and lung injury [15]. Current studies also suggest that CGA has the potential to mitigate cyclophosphamide-induced IC bladder damage and alleviate IC symptoms by reducing inflammation and apoptosis [16]. However, the effect of CGA and its ability to protect against interstitial edema and bladder inflammation has not been adequately addressed in existing literature.

Therefore, the present study aimed to investigate the therapeutic effect of CGA in an LPS-induced inflammatory and edema IC mice model and unravel its impact on voiding behavior. In this study, we demonstrated that administering a specific dosage of CGA significantly improved micturition conditions, alleviating bladder edema and inflammation in LPS-induced mice with IC. This conclusion is supported by substantial evidence from CMG measurements, western blot, histological examinations, and immunohistopathological observations. Our research unveils the previously unexplored therapeutic role of CGA in mitigating bladder inflammation.

## 2. Results

### 2.1. Low CGA Administration Changes the Voiding Function in IP Injection LPS-Induced IC Mice

**Figure 1:**
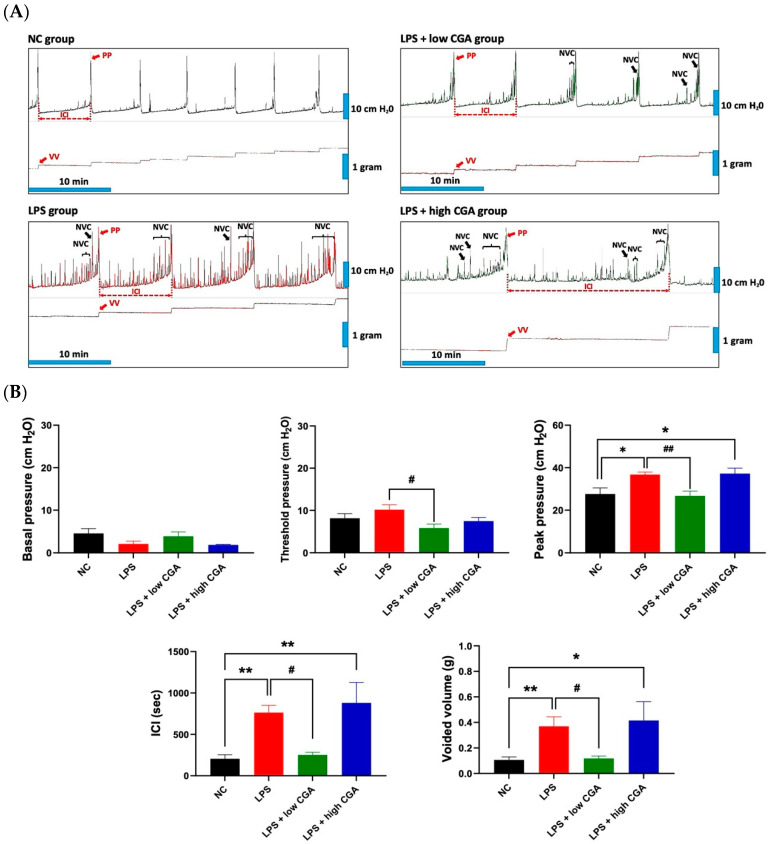
(**A**) Depicts micturition cycle patterns for different mouse groups: Normal Control (NC), lipopolysaccharide (LPS), LPS + low CGA, and LPS + high CGA. Cystometric recordings present micturition pressure, frequency, voiding episodes, and non-voiding contractions (NVC). Lower trace recordings illustrate voiding volume per urination event, offering insights into distinct micturition cycle patterns. (**B**) Provides a comprehensive representation of cystometric parameters, including basal pressure, threshold pressure, peak pressure (PP), intercontraction interval (ICI), and voided volume (VV). Parameters were computed for NC, LPS, LPS + low CGA, and LPS + high CGA groups. LPS + low CGA—(60 µg/mL); LPS + high CGA—(750 µg/mL). Mean values with corresponding standard errors of the mean (SEM) are presented, indicating significant differences (* *p* < 0.05, ** *p* < 0.01, ^#^ *p* < 0.05, ^##^ *p* < 0.01). This visual aid offers valuable insights into variations in cystometric parameters among the experimental groups (*n* = 8 animals/group).

Figure 1A presents a comprehensive and comparative analysis of CMG measurements within the normal control (NC) and experimental groups. The LPS group exhibits significantly longer intercontraction intervals (ICI) compared to the NC group, accompanied by a high frequency of non-voiding contractions. LPS induces a notable increase in micturition pressure and voided volume, along with a decrease in voided frequency compared to the NC group. The LPS + low CGA group shows a significant decrease in threshold pressure, peak pressure, ICI, and voided volume compared to the LPS group. Conversely, the LPS + high CGA group displays elevated micturition pressure and a notably longer ICI duration compared to both the NC and low CGA groups (Figure 1B).

These results demonstrate the normalization of urinary parameters after low CGA administration, highlighting the beneficial effect of low CGA in regulating voiding when the bladder is subjected to external stimulation.

### 2.2. Low CGA Administration Attenuates Bladder Interstitial Edema and Inflammation in LPS-Induced IC

**Figure 2:**
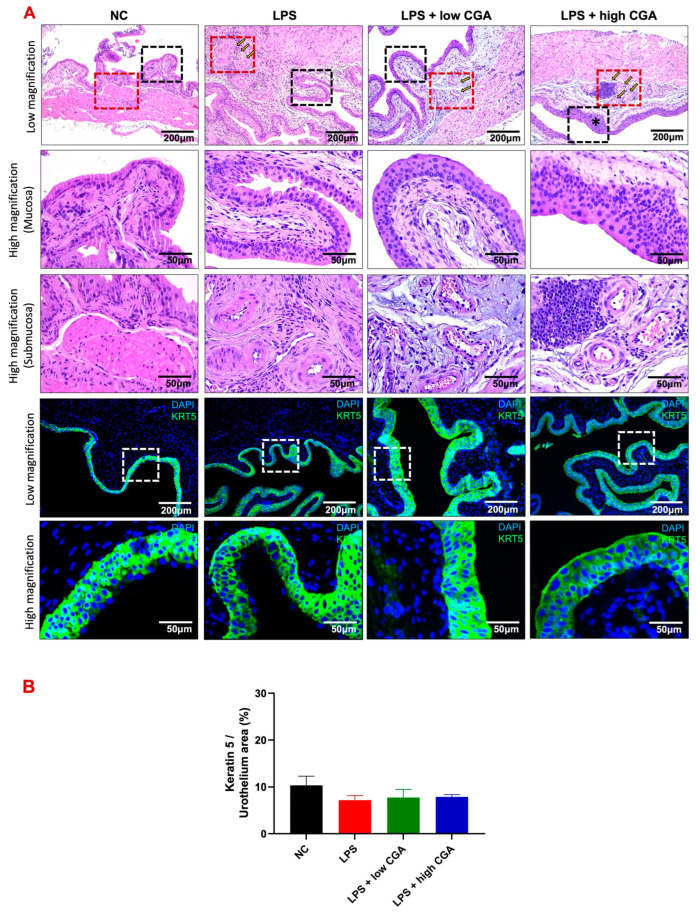
(**A**) H&E-stained and immunofluorescent staining patterns of bladder sections from various mouse groups: normal control (NC), lipopolysaccharide (LPS), LPS + low CGA, and LPS + high CGA. Compared to the NC group, the LPS group displays inflammation in the submucosa (indicated by the red-delineated area) and a thickened urothelial layer (indicated by the black-delineated area) due to inflammation (the black (urothelium) and red (submucosa) delineated areas were correspondingly magnified). In contrast, the LPS + low CGA group exhibits no alteration, manifesting as a normal phenotype of the urothelium attributed to CGA-mediated recovery. Conversely, the * symbol denotes the LPS + high CGA group, which exhibits partial abnormal thickening of the bladder urothelium but shows no significant difference compared to NC. Keratin-5 (K5) staining visualized cytoplasmic intermediate filament proteins within urothelium tissues (white delineated area), with no significant damage detected in the urothelium compared to the NC group. (**B**) Quantification results of K5 indicate that LPS-treated mice have shown no significant damage detected within urothelial tissues (the white delineated area was magnified correspondingly). LPS + low CGA—(60 µg/mL); LPS + high CGA—(750 µg/mL); Black dotted square: high magnification of mucosa; red dotted square high magnification of submucosa). Magnification: Low: 50, high: 100× and higher: 200×; Scale bar: 100 and 200 μm; *n* = 8 animals/group.

To assess interstitial edema and inflammatory status in experimental animals, bladder tissues were collected and subjected to Hematoxylin and Eosin staining (H&E) and immunofluorescence for cytokeratin 5 (K5) expression (Figure 2A). H&E staining revealed pronounced bladder interstitial edema, inflammation, and abnormal morphology in the LPS treatment group, indicative of the impact of LPS induction. Remarkably, the LPS + high CGA group exhibited abnormally thickened urothelium, accompanied by a significant influx of inflammatory cells, including neutrophils and macrophages. Conversely, the LPS + low CGA group demonstrated minimal changes in bladder tissue layer structure, maintaining a normal urothelial phenotype with a slight relief in edema and inflammation. Immunofluorescent staining, particularly cytokeratin-5 (K5) staining, highlighted the presence of cytoplasmic intermediate filament proteins in urothelial tissue. Comparative analysis indicated a tendency for urothelial thinning across all groups compared to the NC group. Interestingly, the LPS + high CGA group exhibited partial damage to K5. Statistical examination, however, revealed no significant differences in urothelial thickness among the groups when compared to the NC group (Figure 2B).

### 2.3. Low CGA Slightly Attenuates Bladder Wall Remodeling after LPS-Induced Inflammatory Bladder

**Figure 3:**
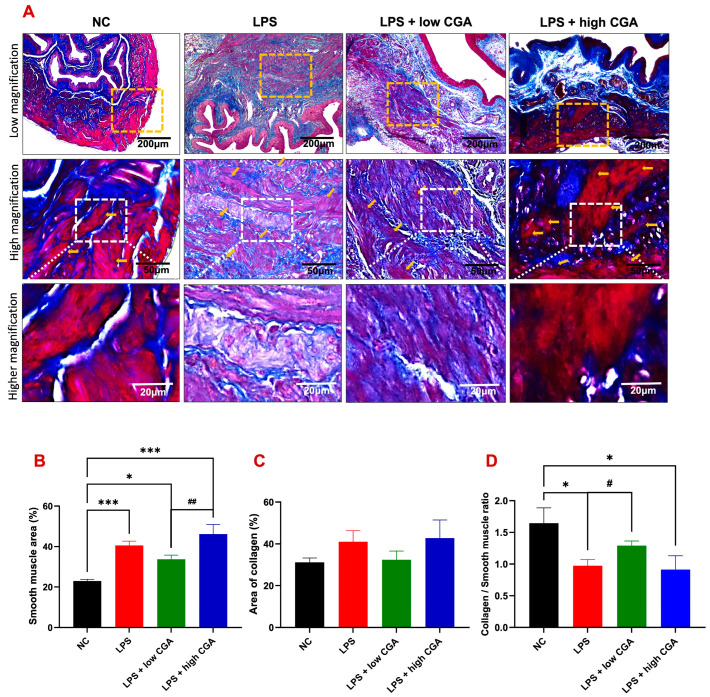
(**A**) Illustrates Masson’s trichrome-stained bladder sections from various mouse groups: normal control (NC), lipopolysaccharide (LPS), LPS + low CGA, and LPS + high CGA. LPS-treated mice exhibited an increased collagen/smooth muscle ratio compared to NC, LPS + Low CGA, and high CGA groups. The LPS + low CGA group displayed evident muscle atrophy and an elevated collagen ratio compared to the NC and LPS + high CGA group. Additionally, the LPS + high CGA group exhibited decreased collagen staining, indicating pronounced inflammatory, fibrotic conditions within the muscle layers (orange-colored arrows), signifying a loss of structural integrity (initially highlighted by the orange color demarcation and further magnified into high magnification with the white-demarcated area, and also further magnified with higher magnification). (**B**) Quantification results enumerate the smooth muscle distribution area, (**C**) collagen distribution area, and (**D**) collagen/smooth muscle ratio in NC, LPS, LPS + low CGA, and LPS + high CGA mice. CGA mice. Yellow dotted square: high magnification of detrusor muscle; White dotted square: higher magnification of detrusor muscle; Scale bar: 200, 50, and 20 μm; Significance level: * *p* < 0.05, *** *p* < 0.001, ^#^ *p* < 0.05, ^##^ *p* < 0.01, *n* = 8 animals/group.

To assess morphometric alterations and structural integrity of the bladder in experimental animals, Masson’s trichrome staining was conducted (Figure 3A). Overall, smooth muscle content exhibited detrusor hyperplasia in all experimental groups compared to the NC group (Figure 3B).

The LPS-induced group revealed irregular muscle skeleton arrangement and a significant augmentation in collagen content in the interstitial layer detrusor muscle layer (Figure 3C). The low CGA group slightly attenuated the pathological development of the bladder wall. In contrast, the LPS + high CGA group presented more severe detrusor hyperplasia and collagen deposition of fibrosis conditions (Figure 3D). These results indicate that a high CGA might have harmful effects on bladder tissue, emphasizing the importance of dosage considerations in treatment applications.

### 2.4. High CGA Administration Causes the Loss of the Superficial Umbrella Cells of the Bladder

**Figure 4:**
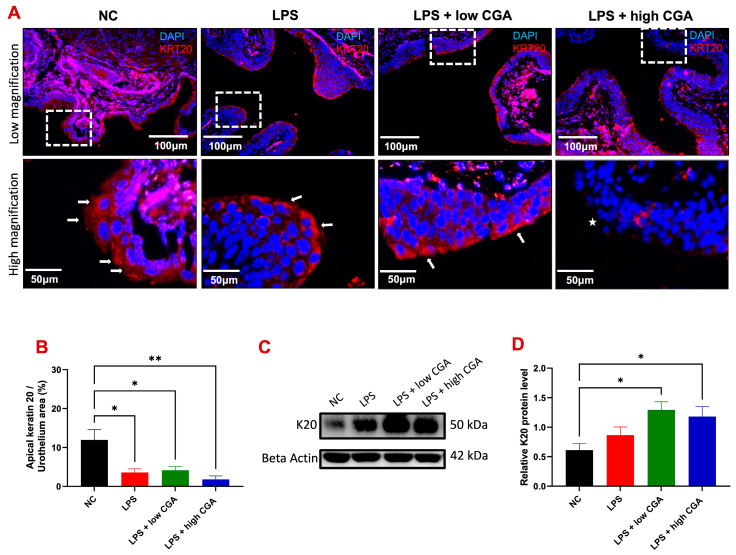
(**A**) Illustrates immunofluorescence staining of the urothelium in mice with bladder barrier-cytokeratin 20 (K20) in various mouse groups: Normal Control (NC), lipopolysaccharide (LPS), LPS + low CGA, and LPS + high CGA. K20 (in red) identifies the superficial epithelium of the bladder (highlighted by the white arrow (K20), indicating urothelial layer recovery with low CGA treatment). Nuclei were stained blue. The * represents the loss of K20. (**B**) Presents quantification results for K20 in bladder tissue. K20 expression decreased in the LPS and LPS + high CGA and low CGA groups compared with NC, and significant differences were noted in the LPS, LPS + high CGA, and low CGA groups. (**C**) Western blotting (total bladder tissue lysates) was used to analyze the protein expressions of K20 between each group, and β-Actin was used as a loading control. (**D**) Quantification of K20 expressed by Western blot analysis exhibited an increase in the LPS and LPS + high CGA and low CGA groups compared to NC, with significant differences noted in LPS + high CGA and low CGA groups. White dotted square: high magnification of urothelium; Scale bar: 100 and 50 μm; level of significance: * *p* < 0.05; ** *p* < 0.01; *n* = 8 animals/group for immunostaining; *n* = 5 animals/group for Western blot analysis.

Cytokeratin-20 (K20) is specifically localized in umbrella cells of the urothelial layer, the initially exposed site to CGA treatment (Figure 4).

Our objective was to investigate if direct exposure of CGA to the bladder has any adverse effects on this crucial defense barrier. In the NC group, an intact apical K20 signal was observed by immunofluorescence, indicating an intact urothelium without apparent damage (Figure 4A). LPS and low CGA treatment showed less K20 expression in mature umbrella cells of the urothelial layer. However, a very weak apical K20 signal in the urothelium was observed in the high CGA group compared to all groups (denoted by *) (Figure 4B). These results demonstrate that the acidic properties of high CGA can adversely affect the bladder mucosa. It needs to be noted that LPS treatment causes a slight increase in total K20 protein in the bladder, but CGA stimulation significantly increases the signal expression of total K20 in the bladder urothelium and lamina propria (Figure 4C,D).

## 3. Discussions

In this study, we assessed the therapeutic impact of CGA in a mouse model of LPS-induced IC. Different doses of CGA resulted in distinct outcomes in the IC bladder. Low CGA administration exhibited a beneficial effect on the regulation of urinary function and delayed the progression of histopathology. However, high CGA administration resulted in prolonged urination time with voiding dysfunction and aggravated injury on histopathological changes. The findings underscore significant differences in bladder function and histomorphometric results among these experimental groups.

Urodynamic evaluation serves as a pivotal diagnostic technique, providing insights into the physiological mechanisms and obstacles impacting the lower urinary tract during the processes of storage and micturition [17]. In our study, intraperitoneal injection of LPS induced an IC mice model that exhibited unique bladder functions, showing pronounced differences and notable similarities to clinical IC symptoms. Clinical patients often experience frequent urination and low urine output. Previous LPS-induced animal models also demonstrated a shortened ICI and less voiding volume in CMG examination, and in our study, the IP injection LPS-induced IC mice revealed a significant decrease in bladder contraction amplitude, consistent with detrusor underactivity symptoms. This result aligns with the findings of Kusakabe et al., 2023 [18], and in our current study, both LPS and LPS with high CGA-treated mouse groups exhibited significantly prolonged ICI and increased urine output compared with the NC group. In contrast, the LPS + low CGA group showed a significant decrease in ICI compared to LPS-treated mice. This result confirms that LPS + low CGA could improve bladder remodeling caused by detrusor insufficiency after LPS induction and restore normal micturition behavior.

Generally, CGA is renowned for its antioxidative, anti-inflammatory, and free radical-scavenging properties [19], which could potentially contribute to its protective effects against IC [16]. Furthermore, our investigation revealed that mice with LPS-induced cystitis, when treated with low CGA, displayed significant alterations in voiding behavior, as evident in cystometric parameters. These changes included decreased voiding frequency, a reduced number of non-voiding contractions, and a decrease in VV, similar to the patterns observed in the NC mice. These observations suggest that a low CGA effectively alleviates clinical symptoms in IC and confers a protective effect against bladder injury while enhancing bladder function in mice [16]. In contrast, LPS + high CGA exhibited noteworthy variations with prolonged ICI and increased VV. These findings suggest that high CGA treatment might not lead to a significant recovery of the IC bladder. Hence, it has not significantly impacted the micturition cycle. This scenario might be due to the afferent nerve projections fulfilling a crucial role in detecting bladder filling and transmitting neuronal signals from the bladder to the brain, thereby regulating urination. The principal bladder afferent pathway is composed of myelinated Aδ fibers and unmyelinated C fibers. It has been widely postulated that Aδ fibers respond to bladder filling under normal circumstances, while C fibers transmit noxious stimuli in pathological situations [20]. The elongated ICI and augmented VV observed might be attributed to the potential bladder interstitial layer inflammation or significant thickening of the detrusor layer caused by the LPS + high CGA. This damage could potentially lead to the suppression of the primary bladder afferent pathway, potentially involving Aδ and C fibers within the IC model induced by LPS or LPS + high CGA.

Inflammation plays a pivotal role in the pathogenesis of IC, with potentially elevated levels of leukocyte infiltration in this condition [16]. Our histological assessment using H&E staining aligns with these findings, revealing a similar pattern of inflammation within the lamina propria of the bladder tissue in the LPS-treated group of mice. Prominent histological changes in IC often encompass bladder mucosal hemorrhage and submucosal edema. Moreover, our observations indicate an increased presence of leukocyte accumulation in the lamina propria, possibly contributing to heightened excitability of primary afferent neurons and thereby fostering bladder hypersensitivity and visceral pain [21]. Besides this, similar to the LPS-treated group of mice, we have observed neutrophil accumulations in the LPS + high CGA group of mice as a host defense mechanism. It has been elucidated that neutrophils play a pivotal role in inflammatory responses, including the generation of reactive oxidative species (ROS) and the release of cytotoxic factors [22]. High CGA treatment might show stronger inflammatory defense mechanisms, though ultimately leading to worsening tissue. In line with our current findings, it has been demonstrated that high CGA stimulates the production of reactive oxygen species (ROS), which, in turn, may activate endothelial cells, leading to the accumulation of neutrophils. This process triggers an inflammatory response mediated by neutrophils through the upregulation of adhesion molecules on both leukocytes and endothelial cells [23]. Consistent with this notion, the observed neutrophil accumulation in the LPS + high CGA group of mice supports the detrimental impact of higher concentrations of CGA.

Masson’s trichrome-stained slides were utilized to assess the composition of bladder tissues. In our current investigation, the experimental group of mice exhibited significant detrusor muscle hyperplasia, resulting in the thickening of the bladder walls. This detrusor muscle hyperplasia was associated with bladder wall remodeling characterized by increased smooth muscle and urothelium, along with decreased collagen. Previous studies have indicated that LPS toxicity may lead to demyelination of the brain and spinal cord [24]. Localized demyelination in the central nervous system is more likely to result in urinary storage symptoms, detrusor overactivity, and compensatory hyperplasia [25].

In the urinary bladder, K20 serves as a marker for umbrella cell differentiation, crucial for maintaining their characteristic shape and integrity [26]. K20 represents a type I cytokeratin of low molecular weight with acidic properties, and its physiological expression is primarily limited to umbrella cells in the normal urothelium [27]. In our current study, K20 expression in the superficial urothelium is partially less in the LPS group. Still, a significant reduction in K20 expression in the umbrella site of the urothelial layer is evident in the LPS + high CGA group of mice, unlike the LPS + low CGA group. This suggests that the low CGA has a positive effect on the urothelial layer, contributing to the repair of umbrella cells. The distribution of K20 was uniformly found in normal umbrella cells, and the reduced expression signifies a depletion of the umbrella cell layer in the LPS + high CGA group [28]. The western blot results also affirm increased K20 expression in the LPS + low CGA group of mice compared to the NC, LPS, and LPS + high CGA groups, pointing to the recovery of the urothelial layer through CGA treatment after LPS-induced damage. A previous study showed an absence of cytokeratin 20 in neuropathic bladders [26], and the possible reason for this might be the impairment of normal voiding function in neuropathy patients, further affecting the expression of differentiation markers like K20. We also found that LPS treatment decreases the total K20 expression, which could impair ultimate K20 differentiation in the urothelium.

## 4. Materials and Methods

### 4.1. Experimental Setup

**Figure 5:**
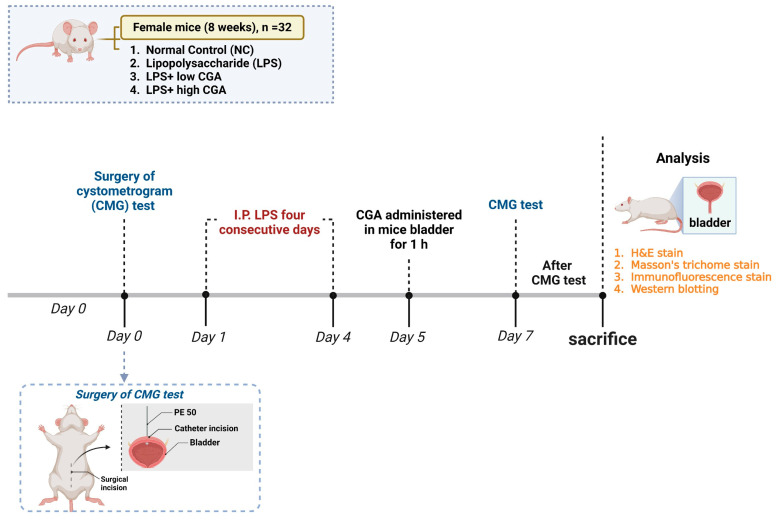
Illustrates experimental setup on a timeline. The total number of animals, *n* = 32, participated in CMG, followed by histopathological and immunohistopathological analyses at scheduled time intervals.

In this investigation, 8-week-old adult C57BL/6J (B6) female mice were employed. The study cohort consisted of a total of 32 mice (*n* = 32), which were equally distributed into four distinct groups (*n* = 8/group): normal control (NC), LPS, LPS + low CGA (low dosage)—(60 µg/mL); LPS + high CGA (high dosage)—(750 µg/mL) (Figure 5).

The allocation was determined based on specific characteristics and varied dosage concentrations. For the experimental group (*n* = 32 mice), intraperitoneal injections of LPS (150 μg/kg) + protamine sulfate (1.5 mg/kg) were administered over four days to induce cystitis. As for the NC group (*n* = 8), saline injections were administered following a schedule akin to that of the experimental animals. Furthermore, varying dosages of CGA (low and high) were introduced directly into the bladder of the specific group of mice. Subsequent experiments, including CMG measurements and histological assessments, were conducted in NC and experimental subjects.

### 4.2. Animal Selection and Housing

In general, female rats are often preferred for micturition studies over male rats for several reasons. To begin with, female rats generally have more stable urinary tract physiology compared to males, as they lack anatomical structures such as the prostate gland and longer urethra present in males, which can introduce variability in micturition patterns. Additionally, female rats have been found to exhibit more consistent bladder contraction responses, making them suitable for studying urinary function and dysfunction. Furthermore, hormonal fluctuations in female rats, particularly estrous cycle changes, can provide insights into the effects of hormonal influences on micturition behavior. Overall, using female rats in micturition studies can help researchers obtain more reliable and reproducible results [29,30,31].

Female B6 (C57BL/6J) mice were procured from Jackson Laboratory, Bar Harbor, ME, USA, for this study. The entire study design and implementation strictly adhered to the guidelines outlined in the Declaration of Helsinki. Furthermore, all research protocols and methodologies underwent thorough review and received explicit authorization from the Fu Jen Catholic University Institutional Animal Care and Use Committee (Approval No.: A 11122, dated: 1 August 2022–31 July 2023). All the mice were housed in the Fu Jen Catholic University animal house, an environment meticulously designed to replicate optimal conditions: a constant temperature of 25 °C, a balanced 12-h light/12-h dark chronobiological rhythm, and an aseptic environment ensuring unrestricted access to both food and water.

### 4.3. Induction of Interstitial Cystitis through E. coli Lipopolysaccharide

Female C57BL/6 (B6) mice were subjected to intraperitoneal injections of LPS obtained from *Escherichia coli* (Sigma-Aldrich, St. Louis, MO, USA) for 4 consecutive days, intraperitoneal injections of LPS (150 μg/kg) + protamine sulfate (1.5 mg/kg) with minor modifications [32]. Similarly, the NC mice were provided with a range of normal saline in a similar manner.

### 4.4. Direct Instillation of CGA into the Bladder

Following a 4-day LPS treatment protocol, the experimental mice received 250 µL of low-concentration (60 µg/mL) and high-concentration (750 µg/mL) CGA injected directly into the bladder from the apex dome. CGA was retained in the bladder for 1 h. This precise delivery was achieved by utilizing a PE 50 tube directly connected with the assistance of a syringe injector. Subsequent to the CGA instillation, a comprehensive analysis of bladder function was conducted, employing CMG measurements. To comply with the Institutional Animal Care and Use Committee (IACUC) guidelines and minimize animal usage, there are limitations on administering the medium dose in the upcoming experiment.

### 4.5. Surgical Procedure for Cystometric Measurements and Bladder Function Evaluation

Prior to the surgery, the mice were anesthetized with Zoletil 50^™^ (tiletamine/zolazepam) and Xylazine (25 mg/kg Z + 7.5 mg/kg X) via intraperitoneal injection. Once the surgical site was shaved, povidone-iodine was employed to cleanse the skin. A precise incision was created along the midline of the body, allowing the bladder to be gently protruded. Utilizing forceps to grip the dome of the bladder, non-absorbable polypropylene sutures (6-0, Prolene, Cornelia, GA, USA) were threaded through the bladder. An additional incision was strategically made near the suture to facilitate the insertion of a polyethylene 50 microtubing catheter into the bladder. The end of the catheter was then heated and molded to create a small cuff. The catheter and bladder are firmly secured with sutures. Subsequently, the catheter is connected to a polyethylene microtube 90, and finally, the muscle and skin layers are sutured closed.

Following the surgical procedure, all the mice were kept in the cage for 1 day for a resting period. Later on, all the mice were subjected to the CMG procedure. For that, the mouse was carefully positioned on the table, and the free end of the PE 50 was latched into the injector machine, initiating the detection of CMG parameters. Using the MP3 pressure transducer (Biopac Systems Inc., Santa Barbara, CA, USA) and Biopac Student Lab 4.1 (Biopac Systems Inc., Santa Barbara, CA, USA) to record data, CMG parameter analysis was performed. The comprehensive range of CMG parameters assessed encompassed basal pressure, peak pressure, threshold pressure, and ICI.

### 4.6. Preparation of Bladder Tissue Homogenates and Western Blotting

Five animals were allotted for the study, and the bladder tissues were shared for the immuno-histological analysis. The methods for tissue homogenates and Western blotting were adapted from Wang et al. (2017) [33] with minor modifications. Mice bladder samples, immersed in 300 μL TPER (78510; Thermo, Waltham, MA, USA) with protease inhibitors (04693132001; Roche, Basel, Switzerland) (*v*/*v*: 25/1), were homogenized using an MP FastPrep-24 5G system (MP Biomedical, Santa Ana, CA, USA). After centrifugation, supernatants were collected, and protein concentrations were determined by the BCA Protein Assay (5000006, Biorad, Berkeley, CA, USA). Western blotting involved 20 μg aliquots, heated, separated on 10% and 15% SDS-PAGE gels, and transferred to PVDF membranes (1620177, Biorad, Berkeley, CA, USA). Membranes were pre-incubated in PBST buffer with nonfat dried milk and then probed with anti-cytokeratin 20 antibody (SC271183, Santa Cruz, Dallas, TX, USA, 1:1000) and anti-Beta-Actin (SC47778, diluted 1:5000, Santa Cruz, Dallas, TX, USA). Secondary antibodies (115-035-003, Jackson Immuno Research Laboratories, West Grove, PA, USA) were applied against anti-cytokeratin 20 and anti-Beta-Actin at 1:5000 and 1:20,000, respectively, before developing blots with Immunobilon Western (WBKLS0500, Merck, Rahway, NJ, USA). Signals were captured using Vision Work (Analytik Jena, Jena, Germany), and data were analyzed by Image Lab software (version 3.0; Bio-Rad, Berkeley, CA, USA) to compare the relative protein abundance of immunoreactive bands. The Western blot analysis was duplicated for accuracy.

### 4.7. Histology Evaluation, Hematoxylin, Eosin (H&E), and Masson’s Trichrome Staining

After CMG testing, the mice were subjected to euthanasia using a high-dose pentobarbital sodium solution. Their bladders were then carefully collected and immersed in a 10% formaldehyde (*w*/*v*) solution for 24 h to ensure preservation. The bladders were subsequently divided into halves and underwent a series of procedures, including dehydration, post-fixation, and embedding in paraffin blocks. Embedding was facilitated by placing a paraffin block in a stainless-steel box on a heated plate set at around 50–60 °C. Liquid wax was used to envelop a bladder specimen positioned at the center, enclosed within a plastic embed box. After cooling and solidifying, any excess wax was removed from the embed box. The embedded tissue was sectioned into 3 µm slices, affixed onto charged slides, and then treated for dewaxing using xylene. The hydrated bladder tissue was subjected to a graduated alcohol treatment (100%, 95%, 90%, 80%, and 70%) and ddH_2_O immersion for 5 min. Subsequent to these procedures, the tissue underwent comprehensive staining using both Hematoxylin and Eosin stain, and Masson’s trichrome as described in our previous study with minor modifications [34].

This investigation included all 32 animals (*n* = 32) in comprehensive histological and immunofluorescence analyses. Each specimen underwent imaging at four specific locations per slide, encompassing two positions on both sides of the intermediate layer of the bladder. Importantly, these designated positions purposefully excluded both the site of surgical catheter insertion and the urethral location. Consequently, the imaging of these positions remained unaffected by the potential influences of surgical catheter insertion and associated sampling procedures.

### 4.8. Immunofluorescence Staining

For immunofluorescence staining, samples were dewaxed and soaked in ethanol for rehydration. Soon after, the slides were left in the blocking solution (10% goat serum, 2% bovine serum albumin, and 0.2% Triton X-100) (Sigma-Aldrich, St. Louis, MO, USA) for 1 h at room temperature. Later, the primary antibodies for anti-cytokeratin 20 antibody (SC271183, diluted 1:100, Santa Cruz Biotechnology, Santa Cruz, Dallas, TX, USA) and anti-cytokeratin 5 antibody (GTX113219, diluted 1:100, Genetex, Irvine, CA, USA), and were incubated with the segments at 4 °C overnight. After the primary antibody treatment, the samples were incubated with a secondary antibody conjugated to Alexa Fluor 594 goat anti-mouse (A-11005, diluted 1:200, Invitrogen, Carlsbad, CA, USA) and Alexa Fluor 488 goat anti-mouse (A11001, diluted 1:400, Invitrogen, Carlsbad, CA, USA) for 1 h at room temperature. For the nuclear colocalization procedure, the DAPI staining method was employed. The analysis and merging of all figures were accomplished through the utilization of ImageJ software Version 1.54g (National Institutes of Health, Bethesda, MD, USA) and Adobe Photoshop (version 25.0) Adobe Systems Inc, San Jose, CA, USA.

### 4.9. Quantitative Assessment

The findings have been represented in terms of means ± standard error mean (SEM). A comprehensive statistical analysis, encompassing one-way analysis of variance (ANOVA) and subsequent Scheffe post hoc examination, was employed to scrutinize disparities among the animal groups. All statistical computations were carried out using SPSS v.18.0 (SPSS Inc., Chicago, IL, USA), with statistical significance established at a threshold of * *p* < 0.05, ** *p* < 0.01, *** *p* < 0.001, **^#^** *p* < 0.05, and **^##^** *p* < 0.01. The study sample size and statistical power were calculated using the SPSS v.18.0 software analysis program. The sample size in all groups achieves a power of above 80% and an alpha value of 5%. For CMG and histological analysis, 8 animals/group (*n* = 32) yielded a statistical power of 0.98; Western blot, 5 animals/group (*n* = 20), showed a statistical power of 0.91

## 5. Limitations of the Study

Presently, CGA treatment has proven to play a pivotal role in restoring acute voiding behavior to normal in lipopolysaccharide inflammation-induced interstitial cystitis in mice. However, our study has limitations. Initially, we focus on acute observations, and the study duration is short. Additionally, exploring a broader range of concentrations and considering alternative administration routes, such as oral, is essential for a comprehensive understanding of the therapeutic potential of CGA. Furthermore, incorporating different models or designing a chronic experiment would further confirm the efficacy of CGA. Since it is a pilot study, further research is warranted to overcome limitations and understand the efficacy of CGA in treating interstitial cystitis.

## 6. Conclusions

This outcome emphasizes that high CGA treatment might not have effectively alleviated the bladder injury caused by LPS, as demonstrated by the significantly reduced expression pattern in the immunohistological analysis. In contrast, low CGA treatment appears to have potentially restored the LPS-induced underactive bladder condition, as evidenced by the normal expression of the detrusor muscle, showing no or less adverse impact after CGA intravesical therapy. In conclusion, our study demonstrated that CGA has the potential to ameliorate LPS-induced bladder injury. However, the direct use of high-concentration CGA in the bladder for treatment may easily lead to damage to the urothelium due to its acidic properties. Future research will assess the impact of different routes of administration and optimize CGA dosage, thereby elucidating its mechanism of action in protecting bladder function and enhancing the potential of CGA in alleviating cystitis.

## Data Availability

The datasets supporting the findings of this study will be shared by the corresponding authors upon request.
